# Inverse‐Designed Nanophotonic Structures for On‐Chip Excitation and Broadband Emission Extraction in Diamond Quantum Spin Sensing

**DOI:** 10.1002/nap2.70264

**Published:** 2026-08-25

**Authors:** Yuqing Zhou, Tsuyoshi Nomura, Yuki Sato, Makoto Nakai, Paul D. Schmalenberg, Ercan M. Dede

**Affiliations:** ^1^ Toyota Research Institute of North America Ann Arbor Michigan USA; ^2^ Frontier Research Center Toyota Motor Corporation Tokyo Japan; ^3^ Toyota Central R&D Labs, Inc. Tokyo Japan

**Keywords:** diamond NV center, excitation, inverse design, light extraction, phonon sideband, photonic integrated circuit

## Abstract

Diamond nitrogen‐vacancy based solid‐state quantum spin sensors on a photonic integrated circuit offer exceptional sensitivity, high spatial resolution, and the potential for substantial device miniaturization. Efficient extraction of broadened phonon sideband emission is particularly important for spin sensing, where measurements rely on fluorescence contrast between spin states rather than indistinguishable photons. However, simultaneous high‐efficiency delivery of excitation light to the nitrogen‐vacancy site and collection of its broad phonon sideband emission remains challenging. This work introduces practical nanophotonic structures using a foundry‐compatible material stack and a fabrication‐constrained inverse design method. An improved regularization scheme enforces minimum feature sizes in both core and cladding. The optimized sensing unit integrates two compact components on a silicon nitride layer: an emitter and a broadband directional coupler. The optimized emitter enhances green (532 nm) excitation while efficiently extracting and coupling broadband red (620–760 nm) emission into a single‐mode waveguide, yielding up to a 3‐fold increase in guided‐mode power relative to a straight ridge waveguide baseline with the same nanodiamond opening. The optimized directional coupler routes ∼80% of the phonon sideband to the detection port while enabling efficient excitation delivery. System‐level simulation and robustness analysis confirm performance and tolerance to nanodiamond placement uncertainty.

## Graphical abstract

Inverse‐designed nanophotonic structures on a foundry‐compatible silicon nitride chip enable efficient in‐plane green excitation and broadband red emission extraction from diamond nitrogen‐vacancy centers. A compact emitter triples the guided optical power, while a directional coupler routes ∼80% of the fluorescence to detection, advancing miniaturized on‐chip quantum spin sensing.

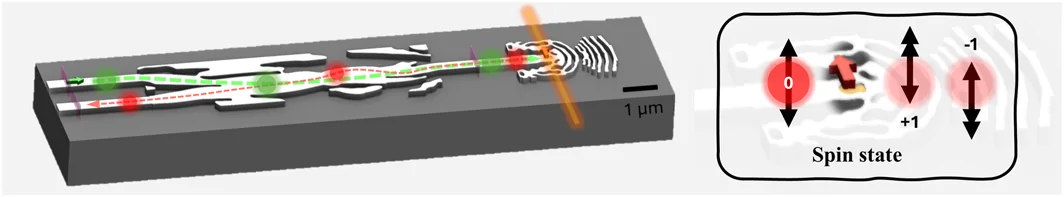

## 1 Introduction

The nitrogen vacancy (NV) center, or color center, in solid‐state diamond material is a photoluminescent defect that exhibits spin‐dependent optical properties. Under green excitation at 532 nm, the NV center emits broadband red fluorescence consisting of a sharp zero‐phonon line (ZPL) at 637 nm and a phonon sideband (PSB) extending to longer wavelengths. The diamond NV center electronic spin state can be controlled using resonant microwave (MW) fields, while an external magnetic field lifts the degeneracy of the ±1 sublevels through the Zeeman effect. Quantum sensors based on diamond NV centers enable detection of magnetic, electric, and temperature fields with exceptional sensitivity and nanoscale spatial resolution under ambient, room temperature conditions [1] in a solid‐state format. Such sensors have been explored for applications including ultrasensitive biomarker detection [2], nanometer‐scale thermometry compatible with living cells [3], GPS‐free navigation [4], and emerging quantum technologies for electric vehicles [5]. These capabilities position diamond NV centers as a versatile technology for advancing applied sensing technologies.

The potential of integrated photonics for quantum technologies [6, 7] is expected to enable the deployment of advanced quantum sensors across diverse industries, driving the miniaturization of optical components and their large‐scale integration. However, photonic chip‐integrated quantum sensors have so far been demonstrated only in limited cases somewhat due to complexity. For example, a quantum sensing system requires the co‐integration of quantum emitters, optical excitation sources, integrated detectors, and photonic components for collection and routing (i.e., waveguides, couplers, and filters), along with MW control structures [8], not to mention any electronics backend and opto‐electronics packaging [9]. Intuition‐driven template designs have been established for on‐chip quantum emitters (e.g., photonic crystals [10, 11]) and routing elements (e.g., parallel directional couplers [12]). The appropriate adjustment of a small collection of dimensional parameters may improve the efficiencies of such components. However, chip‐scale nanophotonic systems increasingly demand broadband operation, multi‐frequency functionality, and compact integration. These requirements make intuition‐driven approaches more complex and ultimately insufficient to meet advanced design objectives. To address these challenges, inverse design methods [13, 14] expand the design space by treating each discretized unit of the design domain as an independent design degree of freedom, and applying continuous relaxations to enable compatibility with gradient‐based optimization.

Existing chip‐integrated quantum emitter designs [15, 16, 17, 18, 19] found in the literature have primarily focused on single‐frequency (e.g., ZPL) implementations, such as Purcell‐enhanced high‐Q cavities, to support quantum applications requiring photon indistinguishability and coherence. However, these designs are not well suited for diamond quantum spin sensing, as they suffer from reduced coupling efficiency and lack the broadband light extraction needed to capture the full NV fluorescence spectrum. Furthermore, previously reported excitation schemes are largely ad hoc, relying either on out‐of‐plane excitation into the photonic chip [19], which does not represent expected in‐plane excitation that would be assumed for a fully integrated photonic circuit, or on a cross‐bar configuration [15] where the excitation channel is oriented orthogonal to the collection waveguide and separated by a gap, resulting in reduced efficiency. For free‐space emission, broadband light extraction has been demonstrated through nanostructuring of bulk diamonds, achieved by inverse design and etching techniques [20, 21]. However, these bulk‐diamond approaches are not directly compatible with integrated photonics, where efficient routing and on‐chip scalability are required. Thus, a chip‐integrated emitter design that simultaneously enables efficient excitation and broadband fluorescence extraction, while remaining compatible with diamond quantum spin sensing, has yet to be demonstrated. Based on this survey, the contribution of this work is twofold. First, the nanophotonic inverse design of a diamond‐based on‐chip quantum emitter is demonstrated to enable notably efficient PSB emission excitation and extraction. Second, this emitter is considered in a larger chip‐scale setup along with associated waveguide routing elements (i.e., coupling and multiplexing) that are further designed to interface with an integrated light source and on‐chip photodetectors plus MW components.

## 2 Methodology

### 2.1 Material Configuration and System Layout

Inspired by earlier demonstrations employing photonic crystal cavities [11, 15] to integrate nanoscale quantum emitters with photonic integrated circuits (PIC), we propose a foundry‐compatible $Si_{3}N_{4}$–on–insulator material system [22]. As illustrated in Figure 1a, a hybrid integration scheme is employed in which nanodiamond NV centers are positioned at deterministic circuit locations via drop‐casting followed by lift‐off processing. To realize excitation and broadband fluorescence extraction from chip‐integrated nanodiamond NV centers, the computational design problem is divided into two stages, as illustrated in Figure 1b. The emitter component is connected to a unified waveguide that simultaneously delivers green (532 nm) excitation light to the quantum material site and collects broadband red (620–760 nm) fluorescence. The unified waveguide further connects to a directional coupler that separates (demultiplexes) the green and red channels, routing them to interface with an integrated green laser source and on‐chip red‐light photodetectors. MW control can be supplied through metal traces, which delivers the MW field to the quantum material site for spin sensing.

**FIGURE 1:**
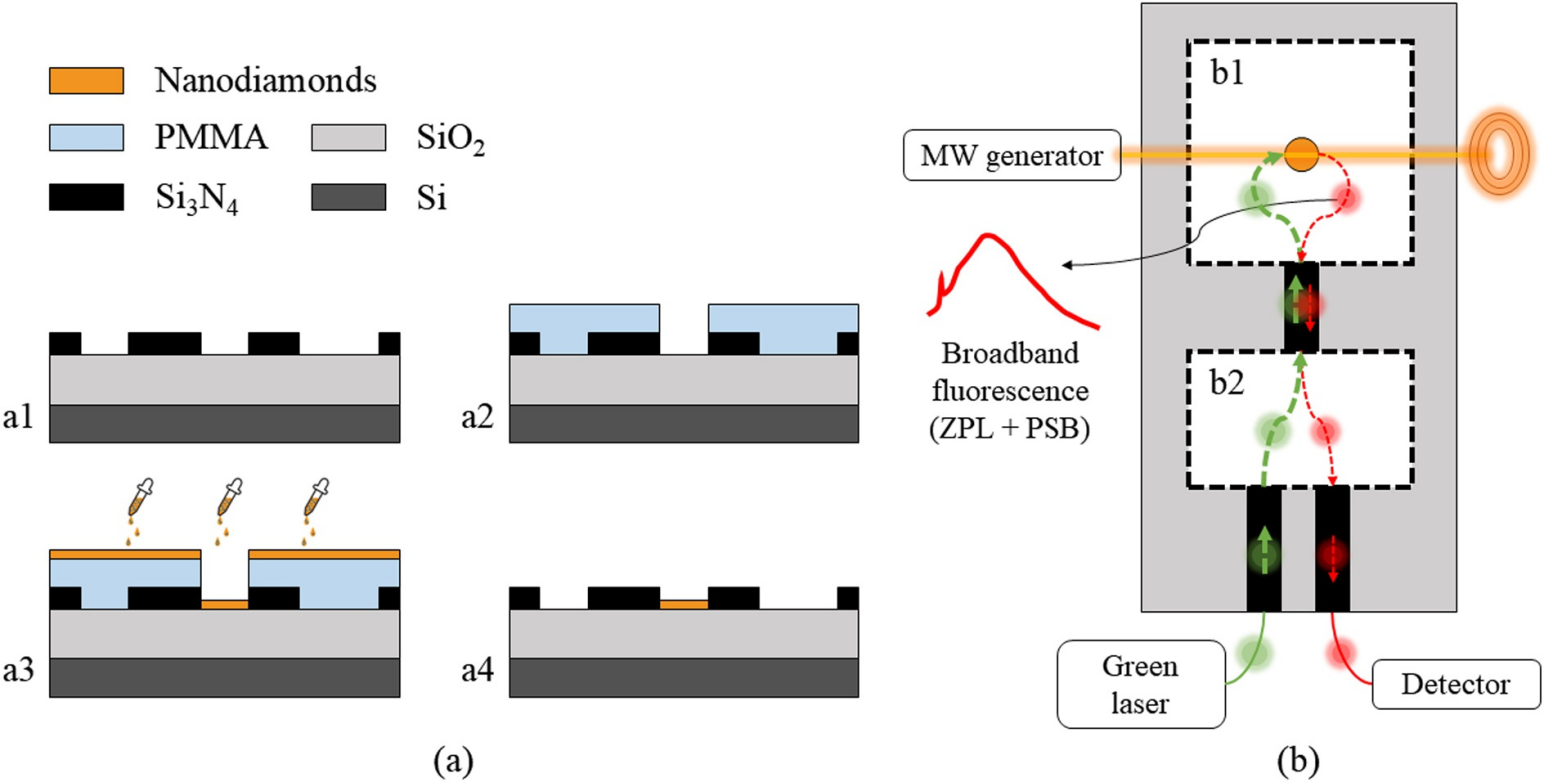
(a) Nanophotonic structure material stacking configuration (cross‐section not to scale) and fabrication workflow including patterning of the designed $Si_{3}N_{4}$ layer (a1), coating of a thin polymethyl methacrylate (PMMA) resist followed by patterning of accurately aligned holes (a2), drop‐casting and drying of nanodiamonds (a3), and lift‐off (a4). (b) PIC conceptual layout (top‐down view) including an *emitter* component (b1), a *directional coupler* demultiplexing component (b2), and other waveguide interconnects.

### 2.2 Fabrication‐Constrained Nanophotonic Inverse Design

Both the emitter and directional coupler components are required to operate over multiple frequencies with broadband performance while also supporting dense on‐chip integration. Given the vast design space, the inverse design process provides advantages over traditional intuition‐driven approaches.

In inverse design, a broad design space is considered by parameterizing the geometric design problem as a material distribution problem via a material interpolation scheme,

(1) $$\epsilon \left(r\right)=\epsilon_{\mathrm{clad}}+\rho \left(r\right)\left(\epsilon_{\mathrm{core}}-\epsilon_{\mathrm{clad}}\right),$$

where $\epsilon_{\mathrm{clad}}$ and $\epsilon_{\mathrm{core}}$ denote the permittivity of the two waveguide cladding and core materials, respectively. The final design variable, ρ∈[0,1], acts as a continuously relaxed parameter for each discretized unit, which describes the nanostructure geometric design.

In order to generate designs that can be fabricated in commercial foundries, the ρ geometry field should be regularized to have sufficiently large minimum feature sizes in both cladding and core and to take only fully binarized values at the end of optimization. Such ρ regularization is implemented through a low‐pass spatial filter, a binarization projection, and an image morphology‐based penalty, applied within a scheduled continuation scheme.

The raw design field ϕ, bounded between 0 and 1, is filtered by a local kernel within a neighborhood set, $N_{i}$, defined by a sizing parameter, R, and kernel weights, $w_{ij}$, as follows:

(2) $${\tilde{\phi }}_{i}=\sum_{j\in N_{i}\left(R\right)}w_{ij}\phi_{j}.$$

Although R is directly related to the minimum feature size specified by a foundry, its value depends on the choice of kernel and typically requires empirical adjustment.

To achieve binarized solutions at the end of optimization, a threshold projection is applied as follows:

(3) $$\bar{\tilde{\phi_{i}}}=\frac{\tanh\left(\beta \tau \right)+\tanh\left(\beta \left(\tilde{\phi_{i}}-\tau \right)\right)}{\tanh\left(\beta \tau \right)+\tanh\left(\beta \left(1-\tau \right)\right)},$$

where τ is the mid‐point thresholding parameter and β controls the strength of the projection. In this work, τ is fixed at 0.5 unless otherwise specified. The projection parameter, β, follows a continuation schedule, starting from 1 and gradually increasing to $\beta_{\text{max}}=20$.

It has been reported [23] that setting τ=1 enforces the minimum feature size in the void phase (i.e., cladding), whereas setting τ=0 enforces the minimum feature size in the solid phase (i.e., core). Intermediate values of τ between 0 and 1 do not enforce a minimum feature size. To enforce the minimum feature size in both the cladding and core regions, additional image morphology‐based operations and an added penalty term to the objective function are proposed. The *dilate* and *erode* operations are defined as a low‐pass spatial filtering (2) followed by a binarization projection (3) with $\tau^{\left(\text{dilate}\right)}=0.2$, $\tau^{\left(\text{erode}\right)}=0.8$, and $\beta =\beta_{\text{max}}$. The *open* operation is defined as an erode followed by a dilate, whereas the *close* operation is defined as a dilate followed by an erode [24]. When the opened and closed geometries are identical, the minimum feature sizes in both cladding and core are satisfied. To promote this condition, a fabrication penalty term, $P_{\text{fab}}$, is formulated as follows:

(4) $$P_{\text{fab}}={\left\|{\bar{\tilde{\phi }}}^{\left(\text{close}\right)}-{\bar{\tilde{\phi }}}^{\left(\text{open}\right)}\right\|}_{\text{RMS}}.$$

The final physical geometry, ρ, used for electromagnetic forward solution is then defined as follows:

(5) $$\rho =\left\{\begin{array}{cc} \bar{\tilde{\phi }} & \text{if}\;P_{\text{fab}}\geq \theta ,\\ {\bar{\tilde{\phi }}}^{\left(\text{close}\right)}\;\text{or}\;{\bar{\tilde{\phi }}}^{\left(\text{open}\right)} & \text{if}\;P_{\text{fab}}<\theta . \end{array}\right.$$

During the early stages of optimization, when the opened and closed geometries differ significantly due to a less aggressive β projection, the standard $\bar{\tilde{\phi }}$ field is used to evaluate the design performance and compute the design sensitivity. As $\bar{\tilde{\phi }}$ becomes more binary and the fabrication penalty, $P_{\text{fab}}$, drops below a threshold, θ, the geometry is switched to either ${\bar{\tilde{\phi }}}^{\left(\text{close}\right)}$ or ${\bar{\tilde{\phi }}}^{\left(\text{open}\right)}$ to ensure accurate enforcement of the minimum feature size in both the cladding and core regions. Since the difference between the two is typically negligible, the selection can be made arbitrarily, although in some cases one may be preferred over the other to avoid undesired local defects. This switching procedure is necessary since $\bar{\tilde{\phi }}$ alone usually does not guarantee compliance with minimum feature size requirements, even when $P_{\text{fab}}$ is sufficiently small. While the individual ingredients of this regularization build upon established topology optimization techniques [23, 24], the key contribution here is the geometry switching mechanism defined in (5). Since a single‐threshold projection with τ=0 or τ=1 enforces the minimum feature size in only one phase, and a small fabrication penalty $P_{\text{fab}}$ alone does not guarantee design‐rule compliance, replacing $\bar{\tilde{\phi }}$ with its opened or closed counterpart once $P_{\text{fab}}$ falls below θ strictly enforces the minimum feature size in both the core and cladding phases simultaneously.

For a given nanostructure geometry described by the permittivity field, ϵ, and a time oscillating current source, J(t), the evolution of the electric field, E(t), and the magnetic field, H(t), can be solved via Maxwell's equations as follows:

(6) $$\begin{array}{cc} \nabla \times E\left(r,t\right) & =-\mu_{0}\;\frac{\partial H\left(r,t\right)}{\partial t},\\ \nabla \times H\left(r,t\right) & =\epsilon \left(r\right)\;\epsilon_{0}\;\frac{\partial E\left(r,t\right)}{\partial t}+J\left(r,t\right), \end{array}$$

where $\mu_{0}$ and $\epsilon_{0}$ are the permeability and permittivity of free space, respectively. From these time dynamics forward solutions, one can formulate a figure of merit (FOM), F, to be optimized and compute its design sensitivity, ∂F/∂ϕ, using the adjoint method combined with the chain rule as follows:

(7) $$\frac{\partial F}{\partial \phi }=\frac{\partial F}{\partial \rho }\;\frac{\partial \rho }{\partial \phi }.$$

The optimized device design can then be obtained via a gradient‐based method following the general optimization formulation for fabrication‐constrained nanophotonic inverse design as follows:

(8) $$\begin{array}{cc} \underset{\phi }{\text{maximize}\;:}\; & F(\rho )-w_{\text{fab}}P_{\text{fab}}(\phi ),\\ \text{subject}\;\text{to}\;:\; & \phi_{i}\in [0,1],\\ \; & \text{design}\;\text{field}\;\text{regularization}\;\phi \;\mapsto \;\rho ,\;(2)-(5),\\ \; & \text{material}\;\text{interpolation,}\;\left(1\right),\\ \; & \text{electromagnetic}\;\text{physics,}\;\left(6\right). \end{array}$$

Here, $w_{\text{fab}}$ is a weighting factor that starts from a small real value and increases progressively with β continuation.

## 3 Results and Discussions

Based on foundry availability and mode confinement across the visible wavelength range of interest, the $Si_{3}N_{4}$ thickness is set to 200 nm, with a standard waveguide width of 400 nm. The finite‐difference time‐domain (FDTD) method is employed to solve Maxwell's equations of classical electromagnetism. All FDTD simulations are performed using Tidy3D [25] from Flexcompute. Gradient‐based optimization iterations are performed using the Adam algorithm implemented in the Optax library [26] from Google DeepMind.

### 3.1 Emitter Component Design

The nanostructured region surrounding the quantum nanodiamond material is defined as the emitter and is designed to focus the excitation light, enhance emission, and couple resulting fluorescence into a waveguide. For the NV emission, the ZPL accounts for only a small fraction of the total fluorescence, typically on the order of 3%–5% at room temperature [27]. The majority of the emission arises from the vibronic PSB. The broadband red PSB target window (620–760 nm) intentionally includes the ZPL at 637 nm rather than selectively suppressing or enhancing it, and the optimization treats the ZPL wavelength no differently from the surrounding PSB wavelengths.

For spin sensing, which relies on fluorescence contrast between spin states rather than photon indistinguishability, the absolute photon count is the critical metric, as it directly enhances the signal‐to‐noise ratio and improves sensing sensitivity.

As shown in Figure 2, efficient broadband red‐light extraction is optimized within a 3μm×2μm extruded design domain by placing an electric dipole source spanning the full NV emission spectrum at the pre‐allocated 200 nm‐diameter opening site. A mode monitor is placed at the waveguide cross section to evaluate the power coupled into the fundamental transverse electric (TE) mode. Since the dipole orientations of drop‐cast nanodiamonds are inherently random, the resulting NV polarizations are uncertain. Prior studies [28] have shown that, owing to spontaneous symmetry breaking, optimizing a single polarization at the expense of others maximizes the mean performance of randomly oriented dipoles. In this work, we select the y‐polarized dipole, which couples most effectively to the fundamental TE mode of the waveguide.

**FIGURE 2:**
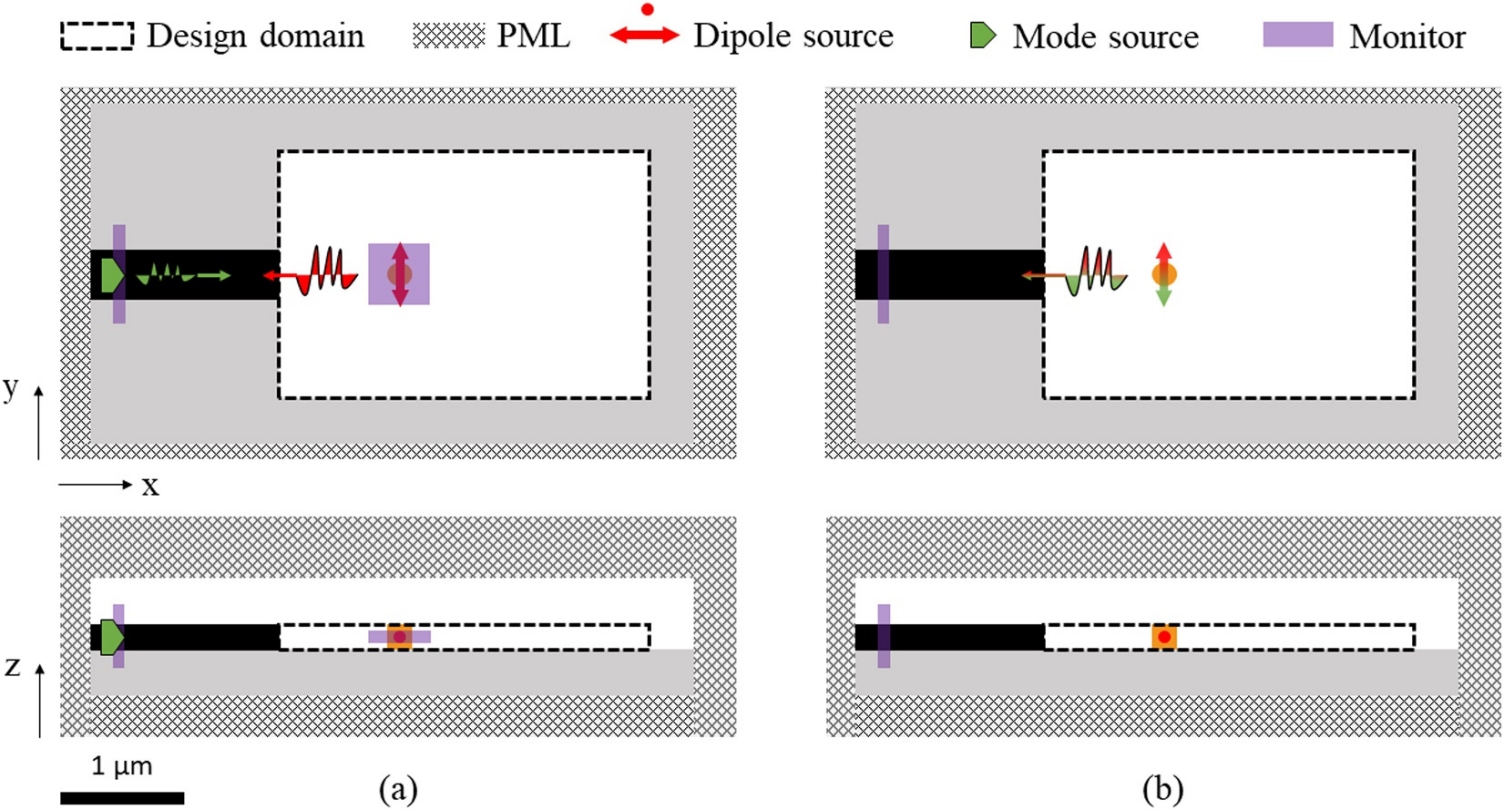
Design domain, boundary conditions, and monitor configurations used to evaluate the FOM, $F_{\text{emitter}}$, for the emitter component inverse design. (a) Original problem setting with separate dipole source for red emission and waveguide mode source for green excitation. (b) Simplified problem setting based on electromagnetic reciprocity where a combined dipole source is assumed to cover both the green excitation and multiple red PSB wavelengths.

The spectrum‐weighted FOM to quantify the degree of emission light extraction is defined as follows:

(9) $$F^{\left(\text{em}\right)}=\sum_{i}w_{\lambda_{i}}^{\left(\text{em}\right)}P_{\lambda_{i}}^{\left(\text{em}\right)}\left(\rho \right),$$

where $w_{\lambda_{i}}^{\left(\text{em}\right)}$ denotes the normalized emission spectral weights [27], which are set to five values (0.1730, 0.2681, 0.2605, 0.1829, and 0.1155) corresponding to the wavelengths, $\lambda_{i}$, of (640.6, 664.1, 682.2, and 720.1, 744.4) nm, for i=1 to 5, respectively. In Equation (9), $P_{\lambda_{i}}^{\left(\text{em}\right)}\left(\rho \right)$ is the design dependent power coupled into the waveguide fundamental TE mode at wavelength $\lambda_{i}$. This power term, $P_{\lambda_{i}}^{\left(\text{em}\right)}$, then accounts for both the source power enhancement provided by the surrounding nanostructure and the coupling efficiency into the waveguide.

To optimize efficient green‐light (532 nm) excitation, the electric field at the quantum material site can be maximized by placing a waveguide TE mode source at the green laser wavelength, as presented in Figure 2a. To reduce computational cost, a single combined FDTD simulation is employed in this work, as presented in Figure 2b, in which the dipole source, $J^{\left(\text{dipole}\right)}\left(t\right)\;\propto \;\cos\left(\omega t\right)$, is broadened to span both the green excitation wavelength and the red (620–760 nm) PSB spectrum. A TE mode monitor is positioned at the waveguide cross section to evaluate both excitation and emission extraction performance. The above approach is justified by electromagnetic reciprocity in the green‐light excitation process.

Note that the refractive index of $Si_{3}N_{4}$ varies by Δn≈0.028 across the 532–760 nm band. The combined single‐broadband‐dipole model, in which the refractive index is held constant at its value at the red PSB center wavelength of 690 nm (n=2.033), is therefore used as a computationally efficient surrogate during optimization. Electromagnetic reciprocity remains valid independently at each wavelength because $Si_{3}N_{4}$ is a linear, reciprocal, dispersive medium, so the sole approximation is the use of a wavelength‐independent refractive index. To assess this simplification, one converged design was re‐evaluated via separate narrow band simulations, each assigning the exact $Si_{3}N_{4}$ index at its wavelength and compared against the combined model. The combined and separate coupling efficiencies, $\eta =P_{\text{mode}}/P_{\text{emit}}$, differ by up to about 5.9%. The end‐to‐end simulation of the assembled system in Section 3.3 instead employs the full wavelength‐dependent dispersive $Si_{3}N_{4}$ model, so the reported system‐level performance already accounts for material dispersion. A fully dispersion‐consistent optimization is possible but requires a dispersion‐specific time‐domain adjoint formulation [29] with auxiliary material variables and additional memory for the dispersive field state [30].

The excitation FOM is then defined as $F^{\left(\text{ex}\right)}=w_{\lambda_{c}}^{\left(\text{ex}\right)}P_{\lambda_{c}}^{\left(\text{ex}\right)}\left(\rho \right)$, evaluated at the center wavelength of the green excitation light at $\lambda_{c}=532$ nm. Therefore, the combined emission and excitation FOM for the emitter component design is summarized as follows:

(10) $$\begin{array}{cc} F_{\text{emitter}} & =F^{\left(\text{em}\right)}+F^{\left(\text{ex}\right)}\\ & =\sum_{i}w_{\lambda_{i}}^{\left(\text{em}\right)}P_{\lambda_{i}}^{\left(\text{em}\right)}\left(\rho \right)+w_{\lambda_{c}}^{\left(\text{ex}\right)}P_{\lambda_{c}}^{\left(\text{ex}\right)}\left(\rho \right). \end{array}$$

The balance between emission and excitation enhancement can be tuned by adjusting the value of $w_{\lambda_{c}}^{\left(\text{ex}\right)}$ in this expression.

The optimized emitter designs presented in Figure 3a–c were obtained using different green excitation weighting, $w_{\lambda_{c}}^{\left(\text{ex}\right)}$, values. All designs exhibit well‐defined geometric boundaries and satisfy a minimum feature size constraint of 80 nm in both the cladding and core regions, owing to the fabrication‐constrained formulation. The emitter performance across the green and red PSB spectra is evaluated from the optical power coupled into the fundamental propagating TE mode of the waveguide, which captures both the local field enhancement at the quantum material site and the mode‐matching coupling efficiency. To achieve efficient one‐way coupling with local field enhancement, the left side of the quantum source is directly connected to the waveguide with carefully shaped boundaries for mode matching, whereas the right side incorporates mirror‐like features that are tuned according to the design priority between the green excitation and red PSB wavelengths.

**FIGURE 3:**
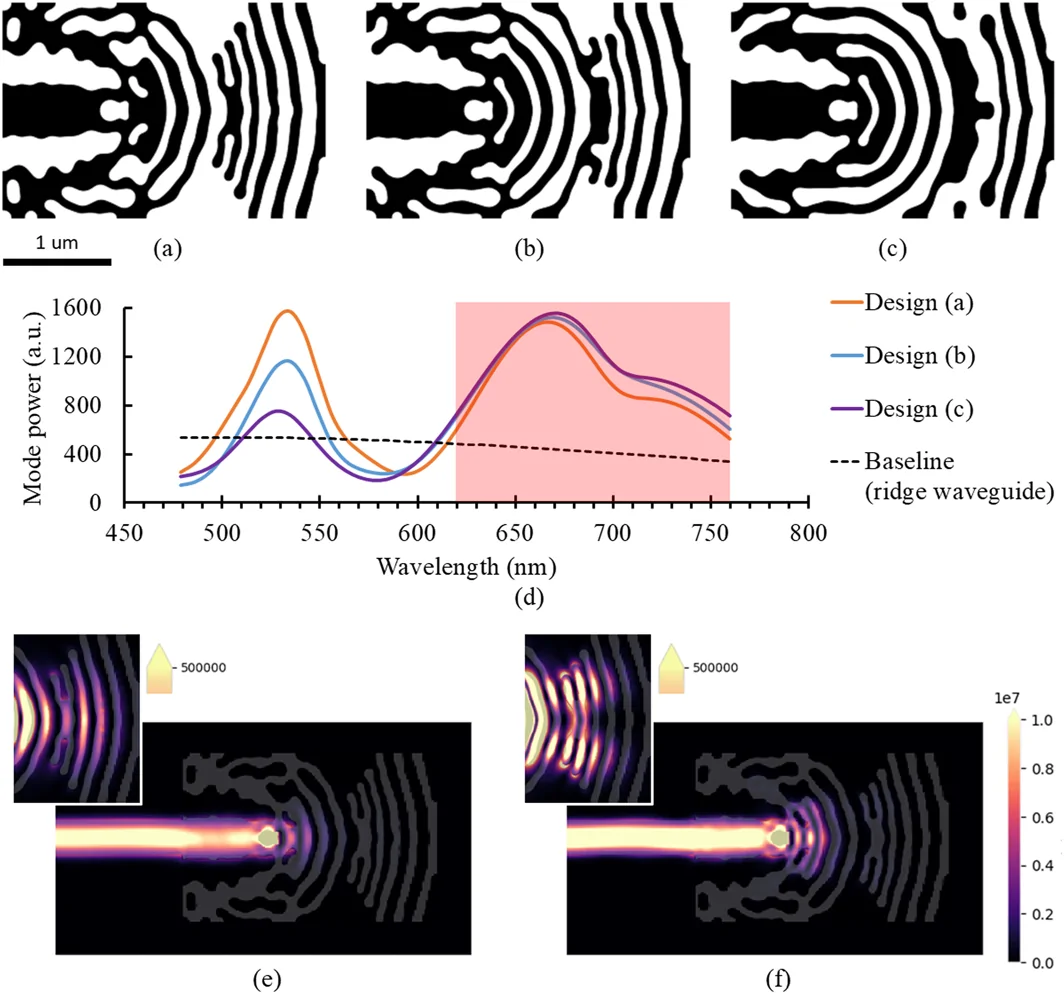
Optimized emitter component designs (a–c), where dark regions indicate waveguide core material while light regions indicate cladding material, and their corresponding power extraction and coupling performances (lower image). The FOM green excitation term weighting factor, $w_{\lambda_{c}}^{\left(\text{ex}\right)}$ in (10), is set to 0.3, 0.2, and 0.1 for (a–c), respectively. The baseline design, dashed line shown in (d), corresponds to a straight ridge waveguide with an opening. (e, f) Simulated electric field energy density, ${\left|E\right|}^{2}$, profiles at wavelengths of $\lambda_{2}=664.1$ nm (second red PSB wavelength) and $\lambda_{c}=532$ nm (green center wavelength), respectively.

In Figure 3d, the dashed line indicates the performance of a baseline design, which is a straight ridge waveguide with an opening.

Design (a) exhibits the greatest enhancement over the baseline design at the green excitation wavelength of 532 nm when $w_{\lambda_{c}}^{\left(\text{ex}\right)}=0.3$. A slight reduction in performance within the red PSB wavelength region is observed compared with designs (b) and (c), corresponding to $w_{\lambda_{c}}^{\left(\text{ex}\right)}$ values of 0.2 and 0.1, respectively. Trade‐offs between green excitation and red PSB performance are expected, as the mirror‐like structures on the right‐hand side inherently favor one wavelength regime over the other. Figure 3e,f present the simulated electric field energy density profiles of design (a) at the selected red phonon sideband wavelength of $\lambda_{2}=664.1$ nm and the green excitation wavelength of $\lambda_{c}=532$ nm. In both cases, efficient one‐way coupling toward the left‐hand side is achieved with proper mode matching, while leakage to the right‐hand side is suppressed by the optimized mirror‐like nanostructure features at the target wavelengths. The inset figures, plotted with a reduced maximum color scale, highlight the wavelength‐dependent utilization of the nanostructured mirrors, whose freeform topologies and shapes contribute differently to confinement at different wavelengths.

Figure 4 shows the iterative optimization convergence of the collected mode power at different wavelengths and their weighted‐sum FOM for design (a). The FOM increases sharply during the initial iterations, then balances among the target wavelengths while satisfying the fabrication constraints, and gradually plateaus upon reaching the maximum iteration limit at iteration 60. Notably, at iteration 30, the final physical geometry variable, ρ, was set (switched) to ${\bar{\tilde{\phi }}}^{\left(\text{open}\right)}$, following the condition in (5), once the fabrication penalty, $P_{\text{fab}}$, fell below the threshold of θ=0.05. As shown in Figure 4c, the design at iteration 29 has small features that persist prior to the switch, underscoring the necessity of the proposed switching mechanism. The transition also introduced minor fluctuations in the FOM values, as seen in the left‐hand side graph.

**FIGURE 4:**
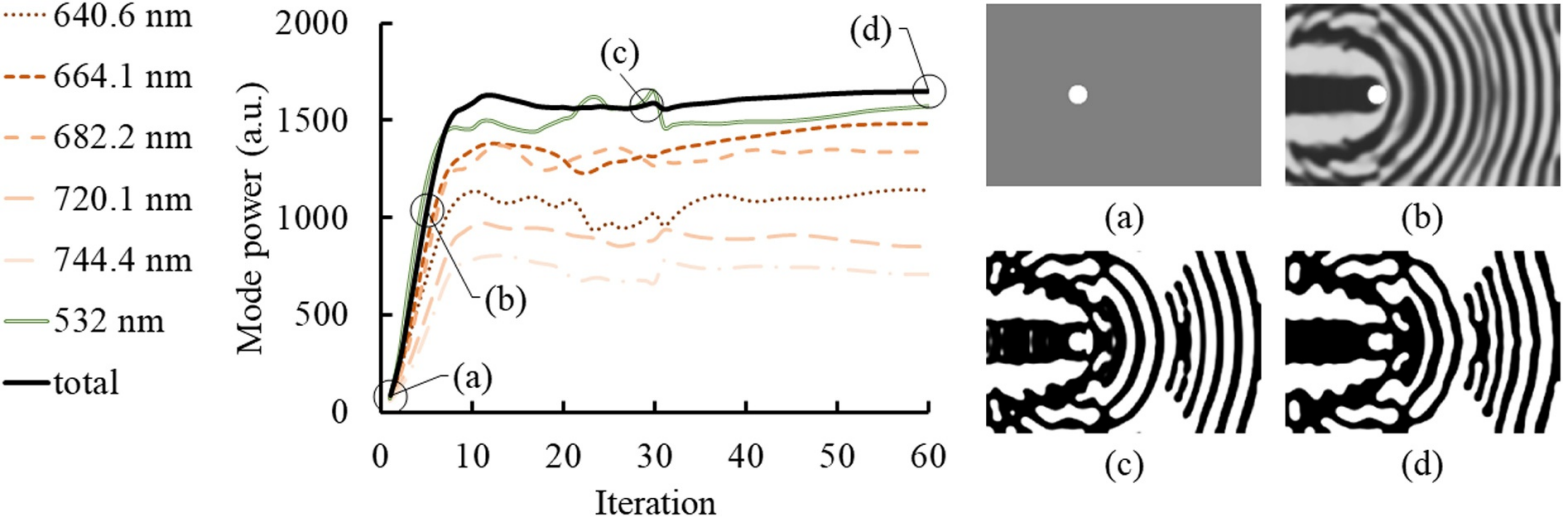
Optimization convergence history (left image) for the emitter component, design (a) from Figure 3. The graph illustrates the convergence and balance of the mode power for the five red PSB wavelengths, $\lambda_{1}$ to $\lambda_{5}$, and the green center wavelength, $\lambda_{c}$, listed from top‐to‐bottom in the key on the left. (a–d) Final physical geometry, ρ, at iterations 1, 5, 29, and 60, respectively; note: dark regions indicate waveguide core material while light regions indicate cladding material.

### 3.2 Directional Coupler Component Design

Since a unified waveguide configuration is employed in the emitter component for both excitation and emission extraction, a directional coupler component is required to separate or demultiplex the green excitation and red emission channels, enabling independent interfacing with a light source and photodetector.

As shown in Figure 5a, efficient separation and routing are optimized within an 8.5μm×2.5μm extruded design domain configured as a three‐port directional coupler. For the red‐emission path, a TE mode source covering the red PSB spectrum can be placed at the unified right port connected to the emitter component, with a mode monitor at the lower left port connected to the detector. For the green‐excitation path, a mode source centered at 532 nm can be applied at the upper left port connected to the laser source, with a mode monitor positioned at the unified right port.

**FIGURE 5:**
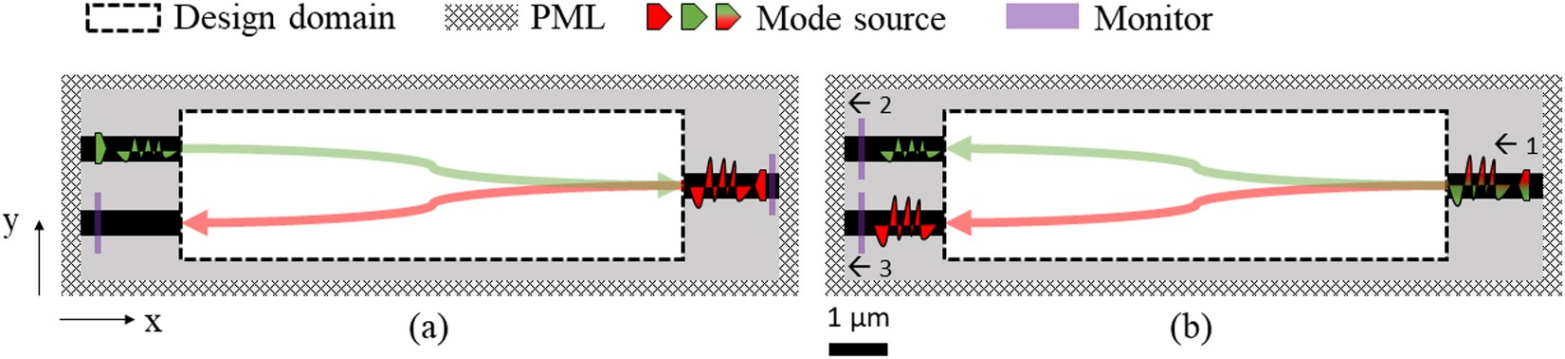
Design domain, boundary conditions, and monitor configurations used to evaluate the FOM, $F_{\text{dc}}$, for the directional coupler component inverse design. (a) Original problem setting with separate green excitation and red PSB mode sources. (b) Simplified problem setting based on electromagnetic reciprocity where a combined mode source is employed that covers both the green and multiple red PSB wavelengths.

To reduce computational cost, a single combined FDTD simulation is used to model both the green and red routes, as presented in Figure 5b. A broadened guided mode source, $J^{\left(\text{mode}\right)}\left(t\right)\propto E_{\text{mode}}^{0}\left(r\right)\cos\left(\omega t\right)$, that spans both the green excitation wavelength and the red PSB wavelength spectrum is used at the unified right port. It is noted that in FDTD, the frequency variation of the mode profile, $E_{\text{mode}}^{0}\left(r\right)$, within the excited bandwidth is neglected. Two mode monitors are placed at the left two ports to evaluate the transmission of the two demultiplexing routes. This simplified modeling approach is again justified by electromagnetic reciprocity in the green‐light excitation route.

The FOM for directional coupler component design is defined as follows:

(11) $$F_{\text{dc}}=\sum_{i}w_{\lambda_{i}}^{\left(\text{em}\right)}{\left|S_{31,i}\left(\rho \right)\right|}^{2}+w_{\lambda_{c}}^{\left(\text{gr}\right)}{\left|S_{21}\left(\rho \right)\right|}^{2},$$

where $w_{\lambda_{i}}^{\left(\text{em}\right)}$ is the normalized emission spectrum weight at wavelength $\lambda_{i}$, as defined previously in Equation (9). The term, ${\left|S_{31,i}\left(\rho \right)\right|}^{2}$, denotes the power transmission for the red‐emission case at the selected red PSB wavelength, $\lambda_{i}$, whereas ${\left|S_{21}\left(\rho \right)\right|}^{2}$ denotes the power transmission for the green‐excitation case. The weighting factor, $w_{\lambda_{c}}^{\left(\text{gr}\right)}$, is newly introduced for the second term on the right‐hand side of Equation (11) associated with the green center wavelength, $\lambda_{c}$, to tune the relative priority of the two routes.

Figure 6a shows the optimized directional coupler design with $w_{\lambda_{c}}^{\left(\text{gr}\right)}$ set to 0.65, while the $w_{\lambda_{i}}^{\left(\text{em}\right)}$ settings follow those used for the emitter component design. As seen in Figure 6b, the design achieves over 80% power transmission at the designated ports and target wavelengths, coupling the green excitation wavelength to port 2 and the broadband red PSB wavelengths to port 3. For comparison, when the entire design domain is filled with the $Si_{3}N_{4}$ core material, the green excitation and red PSB power transmissions drop below 20% and 40%, respectively, at both ports 2 and 3. Figure 6c,d presents the simulated electric field energy density profiles at $\lambda_{2}=664.1$ nm within the red PSB spectrum and at the green excitation wavelength of $\lambda_{c}=532$ nm, highlighting the intended routing paths for the red and green light channels. Notably, unlike standard parallel directional couplers that rely on evanescent field overlap for power transfer and target single wavelength demultiplexing, the inverse‐designed component operates through adiabatic mode conversion to demultiplex the green excitation wavelength and the broadband red PSB wavelengths. This inverse design strategy offers clear advantages, as conventional parallel couplers require long coupling lengths and exhibit limited broadband performance, thereby constraining compact, high‐efficiency integration.

**FIGURE 6:**
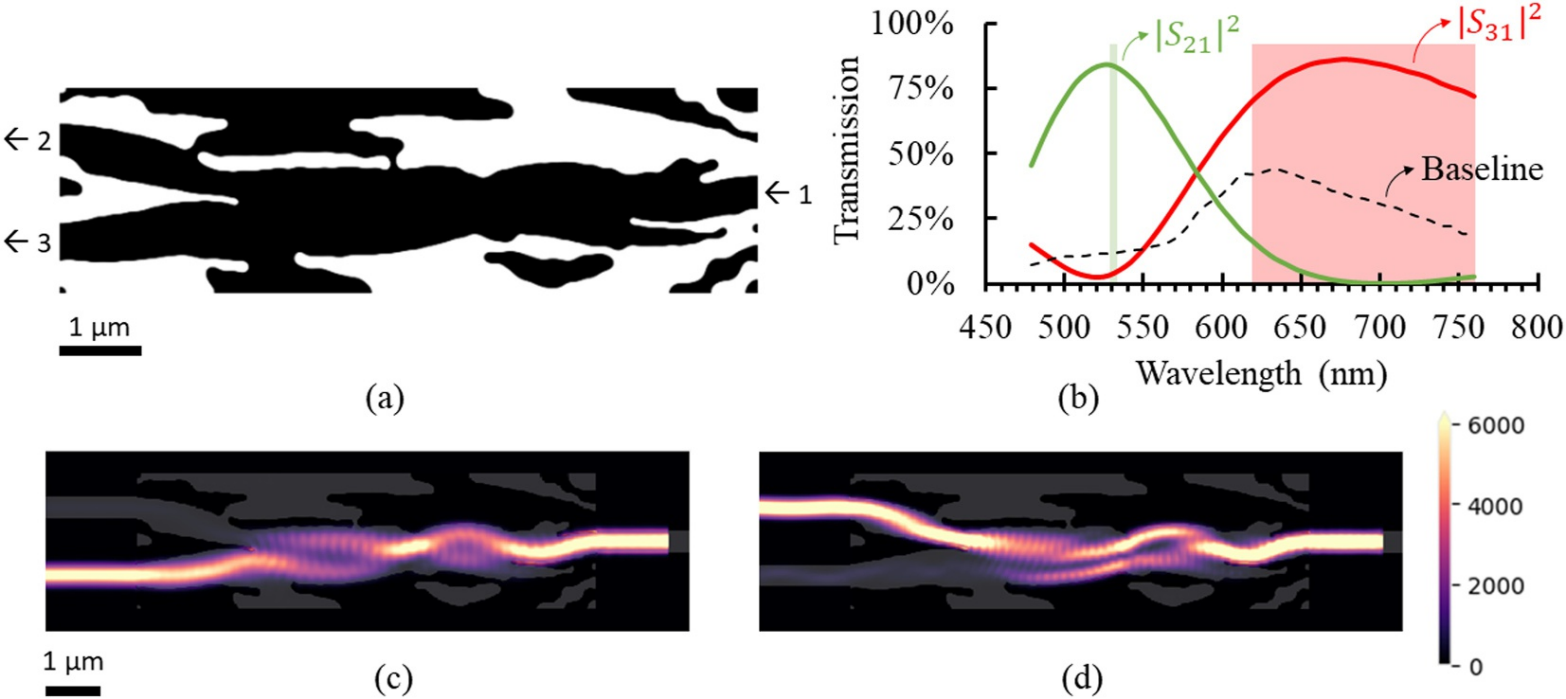
(a) Optimized directional coupler design, where dark regions indicate waveguide core material while light regions indicate cladding material. (b) Power transmission for the green excitation and broadband red PSB routes. The baseline design has the entire design domain filled with the $Si_{3}N_{4}$ core material resulting in ${\left|S_{21}\right|}^{2}$ = ${\left|S_{31}\right|}^{2}$ due to symmetry. (c, d) Simulated electric field energy density, ${\left|E\right|}^{2}$, profiles at wavelengths of $\lambda_{2}=664.1$ nm (second red PSB wavelength) and $\lambda_{c}=532$ nm (green center wavelength), respectively.

### 3.3 System Performance, Robustness, and Uncertainty Analysis

To evaluate the design at the system level in a unified waveguide setup, the inverse‐designed component geometries were converted to GDS format and imported into end‐to‐end FDTD simulations of the assembled system. Wavelength‐dependent dispersive material models were employed. The green wavelength excitation and broadband red PSB performances were evaluated in two independent FDTD simulations using a waveguide mode source and a dipole source, respectively. As shown in Figure 7a, three ports are defined to assess the overall system performance. Figure 7b shows efficient power transmission of approximately 80% from the green excitation source at port 2 to the unified waveguide at port 1, consistent with the component‐level directional coupler results reported earlier. The green light reflections at 532 nm from the emitter component back into the unified waveguide port 1, the green source port 2, and the red PSB detection port 3 are measured as −14.19, −15.60, and −26.65 dB, respectively. Such low back‐reflections are critical for minimizing parasitic feedback into both the laser source and the detector. Figure 7c shows the mode power coupled into the unified waveguide port 1 and the red‐emission detection port 3 from a dipole source located within the opening that hosts the quantum material. The mode power spectrum across the red PSB wavelengths are consistent with the emitter component‐level results and align well with the reported NV emission spectrum [27], demonstrating the effectiveness of the weighted multi‐objective optimization. The power transmission ${\left|S_{31}\right|}^{2}$ within the red PSB wavelength range is approximately 80%, in agreement with the directional coupler component‐level performance. The simulated system‐level electric field energy density profiles, shown in Figure 7d,e, confirm the intended coupling and routing functionalities for both the red PSB spectrum and green excitation optical paths.

**FIGURE 7:**
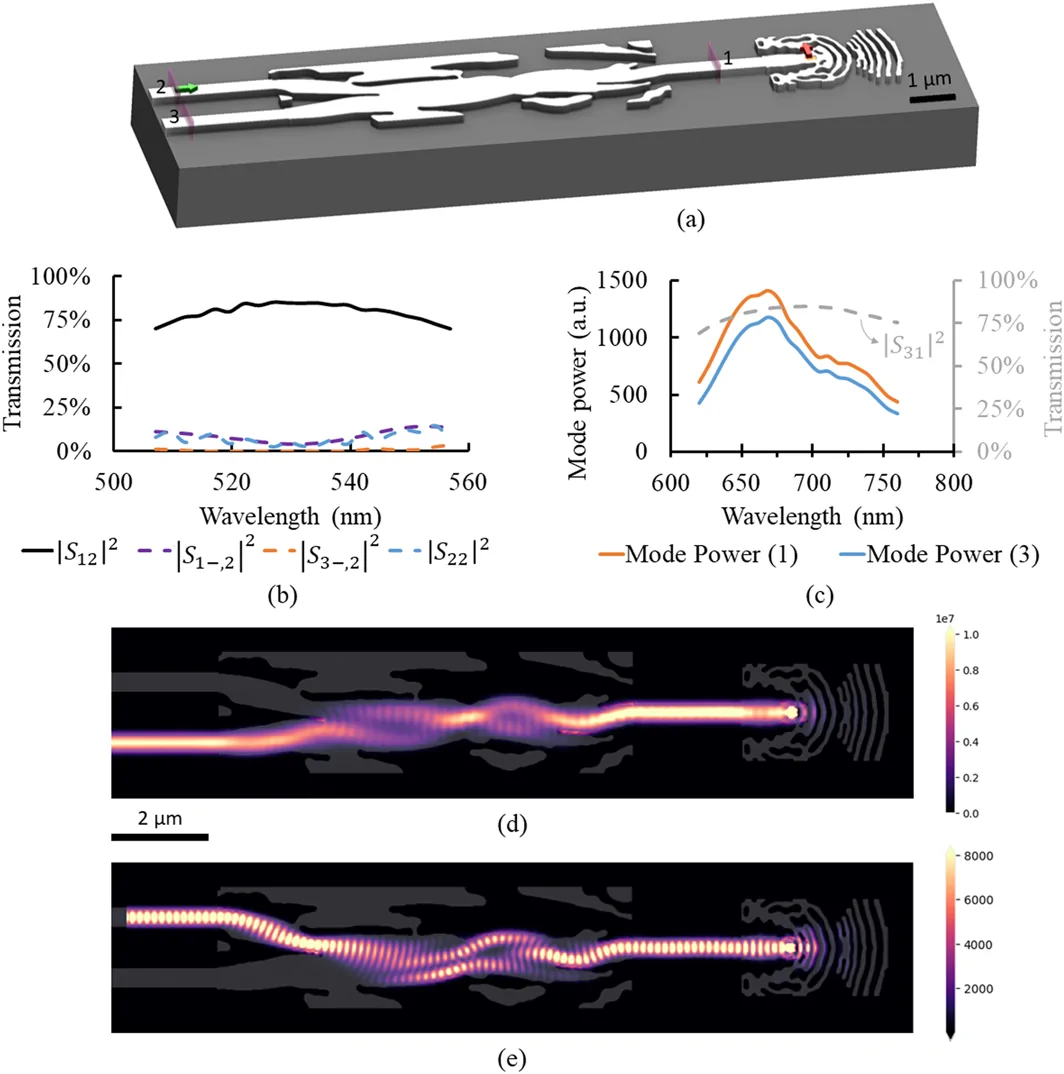
System‐level performance simulation. (a) Imported GDS geometries of the inverse‐designed emitter—design (a) and directional coupler components, including sources and monitor ports used in the system‐level FDTD simulations. (b) Power transmission efficiencies at different ports for the green excitation mode source. (c) Emission extraction, mode power coupling, and transmission efficiencies for the red PSB dipole source. (d, e) Simulated electric field energy density, ${\left|E\right|}^{2}$, profiles at wavelengths of 669 nm (within the red PSB spectrum) and 532 nm (green center wavelength), respectively.

To complement the relative mode‐power comparisons with intrinsic performance measures, Figure 8 reports absolute emission and excitation metrics extracted from the end‐to‐end system simulation. In Figure 8a, the coupling efficiency at port 1 denotes the fraction of the total dipole emission captured into the fundamental guided mode of the unified waveguide (i.e., the on‐chip β‐factor), whereas the collection efficiency at port 3 denotes the fraction that ultimately reaches the red‐emission detection port after passing through the directional coupler. Across the red PSB spectrum, the coupling efficiency ranges from 28.4% to 65.5%, peaking near 670 nm, and the corresponding collection efficiency ranges from 19.5% to 56.0%, with the difference governed by the broadband transmission of the directional coupler. Figure 8b shows the green pump excitation enhancement at the NV site, quantified as the local electric field energy density, ${\left|E\right|}^{2}$, relative to that of the bare ridge waveguide baseline design. The excitation enhancement reaches approximately 2.3× near the 532 nm center wavelength, confirming that the design provides genuine gains in both emission extraction and pump delivery.

**FIGURE 8:**
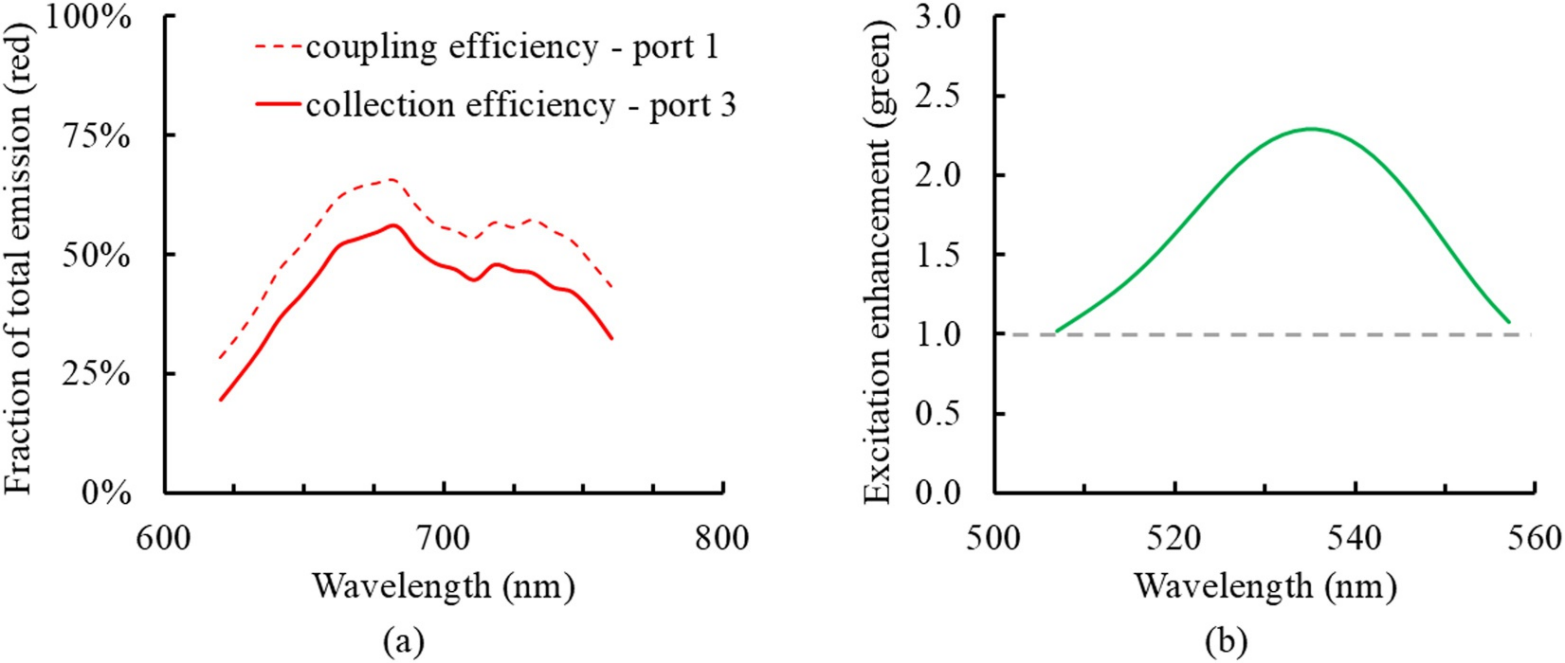
Absolute performance metrics extracted from the end‐to‐end system simulation. (a) Red PSB emission expressed as a fraction of the total dipole emission: the coupling efficiency into the fundamental guided mode at the unified waveguide (port 1, dashed) and the on‐chip collection efficiency at the red‐emission detection port after the directional coupler (port 3, solid). (b) Green pump excitation enhancement at the NV site, quantified as the local electric field energy density, ${\left|E\right|}^{2}$, relative to the bare ridge waveguide baseline design (dashed line).

The robustness of the design to uncertainties associated with nanodiamond drop‐casting is further evaluated by shifting the dipole source away (by ±25–50 nm) from the ideal location assumed during optimization. Figure 9a shows the emission extraction and coupling performance across the red PSB spectrum under such positional uncertainty for the nanodiamond material. As the dipole source is displaced along the horizontal (x‐axis) direction, the collected mode power within the waveguide redistributes across the red phonon sideband spectrum, while the total integrated power over the bandwidth remains high, ranging from 83.7% to 106.7% of the nominal case. In contrast, negligible change is observed when the dipole source is shifted along the vertical (y‐axis) direction, indicating strong tolerance to vertical misalignment of the template‐deposited nanodiamond material. As shown in Figure 9b, the directional coupler component exhibits insensitivity to dipole positioning deviations, with power transmission across the red PSB spectrum remaining stable under both horizontal (x‐axis) and vertical (y‐axis) shifts.

**FIGURE 9:**
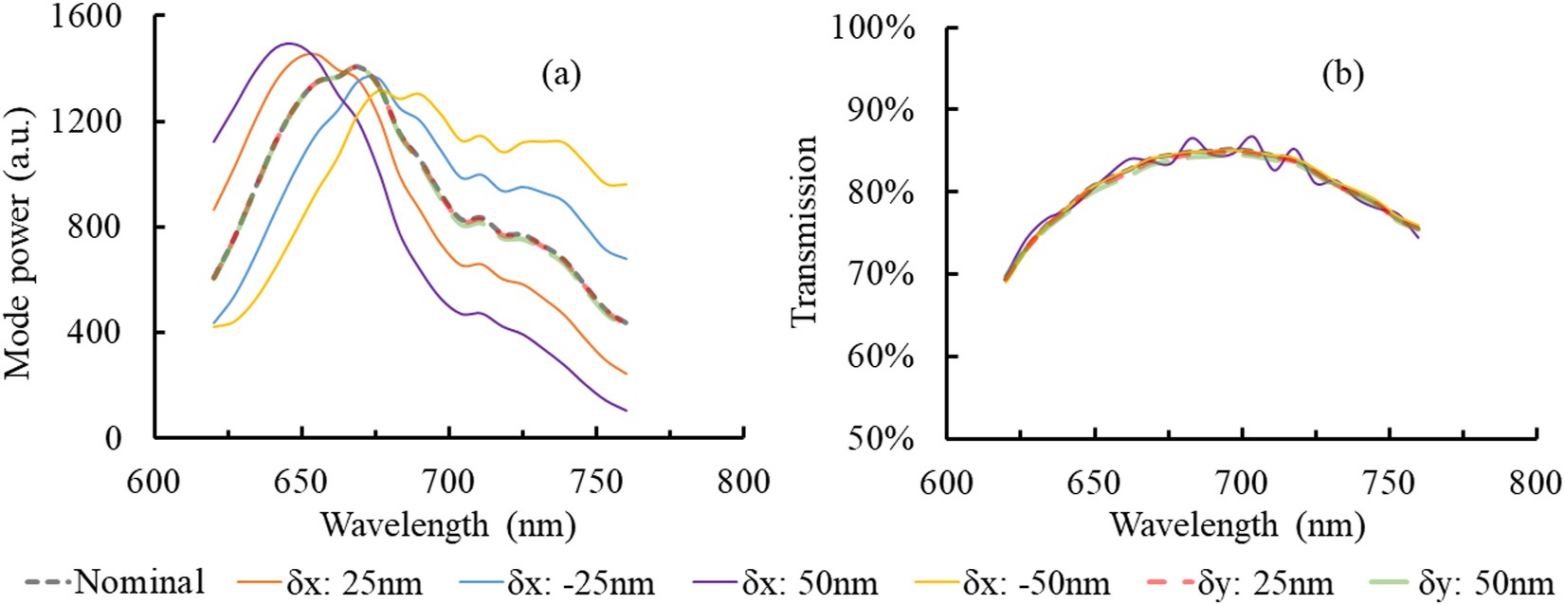
Robustness of the emitter and directional coupler designs to dipole position deviations. (a) Red PSB emission extraction performance for horizontal and vertical dipole source dislocations. (b) Red PSB power transmission efficiency of the directional coupler under the same position deviations.

Since the NV centers in drop‐cast nanodiamonds adopt random orientations, the emission extraction performance is further evaluated as a function of dipole polarization. As shown in Figure 10, the mode power coupled into the fundamental TE mode of the unified waveguide is computed across the red PSB spectrum for the three principal dipole orientations ($E_{x}$, $E_{y}$, and $E_{z}$). The y‐polarized dipole, aligned with the dominant transverse field of the fundamental waveguide mode, couples two to four orders of magnitude more strongly than the x‐ and z‐polarized dipoles. The z‐polarized (out‐of‐plane) dipole predominantly excites TM‐like modes and therefore couples weakly into the collected TE mode, whereas the x‐polarized (longitudinal) dipole radiates weakly along its own axis and overlaps poorly with the transverse mode profile.

**FIGURE 10:**
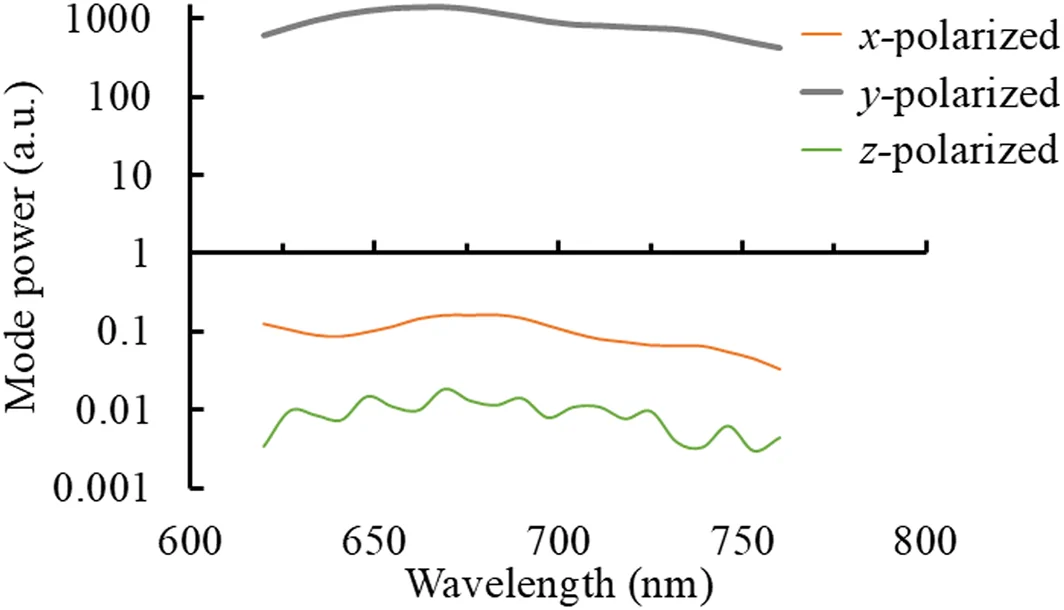
Robustness of the emitter design to nanodiamond dipole orientation. Red PSB emission extraction, quantified by the mode power coupled into the fundamental TE mode of the unified waveguide, for the three principal dipole polarizations ($E_{x}$, $E_{y}$, and $E_{z}$). The y‐polarized dipole couples several orders of magnitude more strongly than the x‐ and z‐polarized dipoles; note the logarithmic vertical scale.

For a single dipole of arbitrary orientation, the contributions from its three principal‐axis components add coherently in the collected mode amplitude. Since the y‐coupling dominates, the extracted power is well approximated by the projection of the dipole onto the optimized (y) polarization. Averaging over a uniform random orientation distribution, the interference cross terms vanish and the expected mean extracted power reduces to one third of the sum of the three principal‐axis powers, which remains dominated by the y‐polarized contribution. This confirms that optimizing a single (y) polarization maximizes the mean performance of the randomly oriented nanodiamond ensemble [28].

The assumed placement tolerances (±25–50 nm) are consistent with reported experimental capabilities of the drop‐cast‐and‐lift‐off scheme. Using a comparable lithographically defined aperture approach, nanodiamonds have been positioned in approximately 70% of the targeted sites to within a similar level of accuracy [15]. Real nanodiamonds may also contain multiple NV centers per particle, and some openings may accommodate more than one nanodiamond. This is compatible with the present design, which targets the absolute collected photon count rather than single‐photon indistinguishability. When single‐emitter operation is required, individual centers within a site could be isolated using frequency‐domain super‐resolution techniques [15], whereas for ensemble‐based sensing, machine‐learning readout can extract magnetic‐field information directly from the collective, randomly oriented ensemble signal [31].

## 4 Conclusion

Based on a practical foundry‐compatible system, this paper presented inverse‐designed nanophotonic structures that simultaneously enabled efficient green excitation and broadband red PSB emission extraction for diamond NV‐center based quantum spin sensing. Minimum feature size control in both core and cladding regions was achieved through an improved image morphology‐based design field regularization scheme. The optimized emitter and directional coupler achieved high extraction and routing efficiencies across the target broadband wavelengths, demonstrating the feasibility of the proposed unified waveguide configuration.

The improved emission extraction efficiency translates directly into enhanced spin‐readout sensitivity. In optically detected NV magnetometry, the shot‐noise‐limited sensitivity improves with the number of detected fluorescence photons. Since the present design increases the fraction of NV emission that is collected into the guided mode and routed to the detector, it raises the detected photon count and thereby improves the magnetic‐field sensitivity. Efficient on‐chip collection with suppressed background coupling further benefits the spin‐readout contrast.

All results reported in this work are computational, obtained from FDTD simulations, and the study is intended as a design‐stage contribution that establishes both the inverse‐design methodology and a concrete, fabrication‐ready device design. The optimized geometries satisfy the minimum‐feature‐size rules of a commercial $Si_{3}N_{4}$ foundry process and have been exported to GDS layouts for tape‐out. Building on this design, additional investigations on chip fabrication and optical characterization are ongoing and will be reported as a separate experimental extension of this research. These investigations also address practical considerations related to nanodiamond drop casting [32] and the intrinsic fluorescence of $Si_{3}N_{4}$ [33].

The computational design framework presented in this paper is broadly applicable to diverse quantum materials, implantation methods, and photonic material systems. Silicon nitride was adopted here primarily because it is widely available in commercial foundry offerings, consistent with the fabrication‐oriented scope of this design study. The intrinsic background photoluminescence of silicon nitride under visible excitation is a recognized consideration for diamond color‐center integration, and recent measurements have begun to separately quantify the core and cladding contributions to this background in integrated waveguides [33]. Should this background prove prohibitive after optical characterization, the same fabrication‐constrained design workflow can be retargeted to alternative lower‐autofluorescence material stacks that have been employed for the integration of diamond and other quantum emitters, such as tantalum pentoxide‐on‐insulator and diamond‐on‐lithium niobate. This work thus establishes a practical pathway toward scalable, high‐performance, and fabrication‐ready integrated photonics for diamond quantum spin sensing.

## Author Contributions

**Yuqing Zhou:** conceptualization, methodology, software, data curation, formal analysis, validation, investigation, writing – original draft, visualization, resources. **Tsuyoshi Nomura:** conceptualization, methodology, investigation, funding acquisition, project administration, resources, writing – review and editing. **Yuki Sato:** conceptualization, methodology, software, investigation, writing – review and editing, formal analysis. **Makoto Nakai:** investigation, writing – review and editing, resources. **Paul D. Schmalenberg:** conceptualization, investigation, writing – review and editing, resources. **Ercan M. Dede:** conceptualization, investigation, resources, project administration, writing – review and editing, funding acquisition.

## Conflicts of Interest

The authors declare no conflicts of interest.

## Data Availability

The data that support the findings of this study are available from the corresponding author upon reasonable request. A public GitHub repository containing the code and data needed to reproduce the results presented in this paper is available at: https://github.com/yuqingz/inverse_design_quantum_emitter.git.
